# The impact of general practitioner morale on patient satisfaction with care: a cross-sectional study

**DOI:** 10.1186/1471-2296-8-57

**Published:** 2007-09-28

**Authors:** Brian McKinstry, Jeremy Walker, Mike Porter, Colette Fulton, Ashley Tait, Janet Hanley, Stewart Mercer

**Affiliations:** 1 Community Health Sciences, University of Edinburgh, 20 W Richmond Street, Edinburgh, UK; 2 Research and Development, Lothian Health, Queens Medical Research Institute, 47 Little France Crescent, Edinburgh EH16 4TJ, UK; 3 Clinical Effectiveness Section, Lothian Health, Stevenson House, 555 Gorgie Road, Edinburgh EH11 3LG, UK; 4 Faculty of Health, Life & Social Sciences, Napier University, Edinburgh, Craiglockhart Campus, Edinburgh EH14 1DJ, UK; 5 General Practice and Primary Care, University of Glasgow, 1 Horselethill Road, Glasgow G12 9LX, UK

## Abstract

**Background:**

The association between stress and morale among general practitioners (GP) is well documented. However, the impact of GP stress or low morale on patient care is less clear. GPs in the UK now routinely survey patients about the quality of their care including organizational issues and consultation skills and the General Practice Assessment Questionnaire (GPAQ) is widely used for this purpose. We aimed to see if there was a relationship between doctor morale as measured by a validated instrument, the Morale Assessment in General Practice Index (MAGPI) and scores in the GPAQ.

**Methods:**

All GPs in Lothian, Scotland who were collecting GPAQ data were approached and asked to complete the MAGPI. Using an anonymised linkage system, individual scores on the MAGPI were linked to the doctors' GPAQ scores. Levels of association between the scores were determined by calculating rank correlations at the level of the individual doctor. Hypothesised associations between individual MAGPI and GPAQ items were also assessed.

**Results:**

276 of 475 GPs who were approached agreed to complete a MAGPI questionnaire and successfully collected anonymous GPAQ data from an average of 49.6 patients. There was no significant correlation between the total MAGPI score and the GPAQ communication or enablement scale. There were weak correlations between "control of work" in the MAGPI scale and GPAQ items on waiting times to see doctors (r = 0.24 p < 0.01). Doctors who perceived that their patients viewed them negatively also scored lower on individual communication, accessibility and continuity of care GPAQ items.

**Conclusion:**

This study showed no relationship between overall GP morale and patient perception of performance. There was a weak relationship between patients' perceptions ofquality and doctors' beliefs about their workload and whether patients value them. Further research is required to elucidate the complex relationship between workload, morale and patients' perception of care.

## Background

General Practice is commonly reported to be a stressful occupation [1-4]. For many doctors it is an accepted part of the job for which they have found coping mechanisms. For others, however, heavy workload compounded by feelings of limited control, self-criticism, balancing home and work demands, or difficulties at work have been associated with high levels of psychological distress [5-7] and it is recognised that prolonged levels of excessive stress may lead to low morale [8]. It seems reasonable to propose that a stressed general practitioner whose morale is low is unlikely to perform well, and that this may lead to patient dissatisfaction.

The Morale Assessment in General Practice Index (MAGPI see additional file 1) [9] is a validated tool designed to assist doctors to determine the areas of their life that may be causing them longer-term stress and gives them the opportunity to compare their own morale score against that of their colleagues. It explores areas including: control of work, support/relationships at work, perceptions of effectiveness as a doctor, home support, and contentment with career choice, health, happiness and alcohol use.

The introduction of the 2004 contract for UK general practitioners provided an opportunity to survey patients' perceptions of their GPs' service. One such, widely used, validated survey instrument, the General Practice Assessment Questionnaire (GPAQ) [10-12], measures three components; patients' perception of practice accessibility; the quality of their consultation; and their assessment of the consultation outcome using a modified version of the Patient Enablement Instrument [13] where lower scores indicate lower levels of 'satisfaction'.

We hypothesised the following:

1. High scoring (low morale) doctors on the MAGPI would be scored lower by their patients on the consultation quality and enablement components of the GPAQ.

2. Components of the MAGPI relating to control of work and support might reflect a practice which was poorly organised and doctors who scored high on these components would score low on the accessibility components of the GPAQ.

3. Doctors who perceived that their patients did not appreciate them (i.e. high score) would score lower on patient assessment of their consultation skills and availability.

## Method

NHS Lothian (in SE Scotland) offers a free service to general practitioners to provide GPAQ forms and to score the forms for them (in return for anonymised use of the collective data). Just over 90% of practices take part in the scheme (the others using an alternative assessment). We wrote to all doctors participating in this scheme to ask if they would be willing to complete a MAGPI questionnaire around the same time (within 4 weeks) as the GPAQ was being collected. Additionally, as there had been no previous test of the stability of the MAGPI over time, the test-retest reliability over 4 weeks was confirmed by asking participants to repeat the MAGPI four weeks later.

Doctors were assured of confidentiality as questionnaires were numbered using a pre-assigned secure system linked to their GP number. All MAGPI forms were posted directly to the research team and linked data from the GPAQ survey was passed from the health board using the same secure number so that no researcher was aware of the identity of any of the participants, nor the health board aware of the doctors' individual MAGPI results.

GPAQ data was collected from sequential patients over the age of 16 years and the adult accompanying a child under 16 year attending doctors in participating practices. On arrival for check in receptionists gave all eligible patients the GPAQ questionnaire and requested patients to complete it *after *seeing the doctor and to deposit the questionnaire in a sealed envelope in a box at the reception desk. Practices were asked to collect 50 questionnaires for each doctor. Each practice was given a limited number of the questionnaires (55 per doctor) to distribute and so strenuous efforts were made to ensure patients completed the questionnaires in the surgery before leaving. This system was designed to ensure that doctors were unable to 'cherry-pick' patients whom the thought might be uncritical.

### Analysis

Test/retest stability of the MAGPI was assessed by calculating the observed difference in MAGPI scores across the two administrations of the instrument (i.e. later score minus earlier score), and performing a one-sample t-test of the null hypothesis that the mean difference score was zero. Spearmann's correlation coefficient was also calculated.

The level of association between GPAQ and MAGPI scores was determined by aggregating the former to the level of the individual doctor (as mean values), and calculating the rank correlation of the aggregated GPAQ 'communication' and 'enablement' scores with MAGPI. Associations between selected individual MAGPI and GPAQ items were also assessed via rank correlation.

### Response rates

475 doctors were approached. 296 (62%) agreed to take part in the main study and 198 provided two valid forms for test-retest reliability

Of those who agreed to take part, an average of 49.6 (SD: 4.4) GPAQ forms were completed. Each participating doctor was allocated 55 forms to be distributed to patients so the overall patient completion rate was 90.2%. As anonymity was assured it was not possible to determine if the participating doctors differed from non-participants.

Twenty doctors with fewer than 48 usable GPAQ responses were excluded from analysis, since it was felt that small numbers of responses did not permit calculation of doctor-level mean scale values to an acceptable level of precision particularly the enablement component of the instrument [13]. On this basis, valid communication and enablement scores were calculated for 276 doctors.

## Results

### Test-retest stability of MAGPI

For the 198 doctors who completed two MAGPI questionnaires, the mean score for the first (earlier) MAGPI was 18.6 (standard deviation: 3.0); the corresponding value for the second (later) administration was 18.2 (SD: 3.0). Spearmann's correlation was 0.85 p < 0.0001. Although the small difference in means was statistically significant (p < 0.01) it is unlikely to represent a clinically significant difference suggesting acceptable test-retest reliability.

### Associations between GPAQ and MAGPI

#### Associations of total MAGPI score with communication and enablement scales of GPAQ

The GPAQ communication and enablement scales yield a range of values from 0 (poorest) to 100 (best). The mean doctor-level communication scale value was 85.1 (SD: 4.9) and average enablement scale was 65.4 (SD: 8.3). These compare with GPAQ national benchmarks of 80 for the communication scale and 65 for the enablement scale [14].

There was no significant rank correlation between total MAGPI score and the GPAQ communication scale (r_s _= -0.03; p = 0.67) or the enablement scale (r_s _= -0.03; p = 0.74). Table 1 also shows no difference in the mean scores of GPAQ communication or enablement scales when analysed by low, medium and high MAGPI scorers.

**Table 1:** The relationship between MAGPI scores and mean GPAQ communication and enablement scores

**MAGPI score range**	**Number of GPs (%)**	**mean GPAQ communication score (95% CI)**	**mean GPAQ enablement score (95% CI)**
0–15	39 (19%)	85.3 (83.6 – 86.9)	65.2 (62.3 – 68.0)
16–17	57 (28%)	84.7 (83.3 – 86.0)	65.5 (63.2 – 67.8)
18–19	40 (19%)	84.5 (82.8 – 86.3)	65.2 (63.0 – 67.4)
20–21	42 (20%)	85.7 (84.2 – 87.3)	64.6 (62.1 – 67.1)
22 and above	29 (14%)	84.5 (83.2 – 85.8)	64.8 (61.1 – 68.4)

#### Associations of individual MAGPI items with GPAQ accessibility items

Table 2 shows that there were weak correlations between the MAGPI items: control of work and work/home life balance with the GPAQ items relating to the time patients waited for a consultation to begin. GPs who reported that they were having difficulty managing their workload and/or who were finding it difficult to keep a balance between work and home life had patients who reported longer times waiting for their consultation to begin, and who rated these longer waiting times as poor.

**Table 2:** The relationship between components of the MAGPI and GPAQ accessibility questions

**MAGPI component**	**GPAQ component**	**Correlation r_s _**=	**p **=
Control of work	How do you rate the way you are treated by receptionists at the practice?	-0.15	0.02
	How do you rate the length of time you have to wait? (*Higher is better)	-0.21	<0.01
Home-life balance	How long do you usually have to wait at the practice for your consultations to begin? (Higher means longer waiting time)	0.24	<0.01
	How do you rate the length of time you have to wait? (*)	-0.20	<0.01
Doctors' perception of what patients think of them	How do you rate ability to get through to the practice on the phone? (*)	-0.17	0.01
	How do you rate the frequency of seeing your usual doctor? (*)	-0.16	0.02
	How do you rate how thoroughly the doctor asked about your symptoms and how you are feeling? (*)	-0.16	0.02
	How do you rate how well the doctor listened to what you had to say? (*)	-0.16	0.02
	How do you rate how well the doctor put you at ease during examination? (*)	-0.15	0.02
	How do you rate how much the doctor involved you in decisions about your care? (*)	-0.17	0.04
	How do you rate how well the doctor explained your problems or treatment? (*)	-0.20	<0.01
	How do you rate the amount of time your doctor spent with you today? (*)	-0.20	<0.01
	How do you rate the doctor's patience with your questions or worries? (*)	-0.16	0.02
	How do you rate the doctor's caring and concern for you? (*)	-0.18	<0.01
	After seeing the doctor today do you feel able to understand your problem(s) or illness? (Higher is better)	-0.15	0.02

Similarly, the doctors who had low perceptions of how they were viewed by their patients was weakly correlated with both poorer scores on all the individual GPAQ communication scale items, and also with the individual items of the GPAQ which explored accessibility and continuity of care. While all the correlations are weak, they are all in the expected direction.

## Discussion

The present study investigated the association between GP moral and patient satisfaction. We failed to find any relationship between morale as measured by the overall MAGPI score and doctor-level patient perceptions of consultation communication or patient enablement. The relationship between doctor morale and patient perceived performance is likely to be complex and so it is perhaps not surprising that the association between them in a cross-sectional study is not powerful. Our study did however, show a weak correlation between doctors' perceptions of their control of work and patients' perceptions of accessibility which we had hypothesised as a marker for heavy workload and/or poor organisation. There was also a weak correlation between doctors' general perception of how patients viewed them and patients' rating of the quality of their consultations. Similar, but rather stronger associations than this have recently been reported between doctors' perceptions of patients' views and patients' rating of consultation quality using aggregated data in a different patient feedback tool [15]. However, in terms of individual consultations there is evidence that doctors are not good at estimating their patients' satisfaction [16].

The study had some limitations. It was restricted to those who volunteered to take part and it is possible that the 38% of GPs who chose not to take part may be different from those who did. Patient participation rates were high, however, and given the way the data collection was organised there was little potential for case selection by GPs. Although the MAGPI showed some loss of stability over time in terms of the test-retest reliability the difference was small and unlikely to affect the outcome. While the weak associations mentioned above were statistically significant, given the large number of possible associations, such "significant" results must be treated with some caution. Of some reassurance was the finding that where significant associations were found they were all in the expected direction (poorer rating by patient associated with poorer rating by doctor of components contributing to morale).

The MAGPI is strongly correlated with the General Health Questionnaire [9,17], but has several components which, while not arising from the doctors' work (such as health and problems in home life) may impact on it. This may explain some of the lack of correlation with practice based factors. Additionally, satisfaction is to a large extent predicated on expectation. Patients long used to what might externally be viewed as poor practice may come to see it as normal, blunting the effectiveness of the GPAQ as an instrument. Our previous work suggested that doctors working in deprived areas were more likely to perceive heavy workloads [9] and a recent national survey showed that GPs in deprived areas in Scotland reported stress and time in consultations as significantly more limiting than GPs in affluent areas [18]. However, good organisation and working relationships within such practices may off-set some of the damage to morale which this may cause [19].

The weak association between GP perceived heavy workload and perceptions of poor service by patients may suggest such workload is bad for both doctors and patients, but we have to remember that these are perceptions of workload. Inefficient doctors may perceive workload to be heavy and also fail to satisfy their patients. Further research is therefore required to compare practices with differing consultation rates per doctor and chronic disease load and also the impact of providing additional resources on patient satisfaction and doctor morale.

## Conclusion

This study showed no relationship between overall GP morale and patient perception of performance. There was a weak a relationship between patients' perceptions ofquality and doctors' beliefs about their workload and whether patients value them. However, firm conclusions about the impact of heavy workload on quality of service cannot be based on doctor self-assessment alone. Further research is required to elucidate the complex relationship between workload, morale and patients' perception of care.

## Competing interests

The author(s) declare that they have no competing interests.

## Authors' contributions

BMcK lead the research and wrote the paper, JW analysed the data and helped write the paper, MP helped analyse the data and helped write the paper, AT and CF helped plan the research collected the data and helped write the paper, JH helped plan the research and helped write the paper, SM Had the idea for the research, took part in discussions about the analysis and helped plan and write the paper.

All authors read and approved the final manuscript.

## Pre-publication history

The pre-publication history for this paper can be accessed here:



## Supplementary Material

Additional file 1 Magpi new. Example of questionnaire used in this study. Click here for file

## References

[B1] Royal College of General Practitioners (1998). Stress and general practice London.

[B2] British Medical Association Scotland (2001). The reality behind the rhetoric: a survey of the views of GPs in Scotland on morale, service provision and priorities for improving primary care Edinburgh.

[B3] Kmietowicz Z (2001). GPs threaten to leave NHS as stress levels rocket. BMJ.

[B4] Sullivan P, Buske L (1998). Results from CMA's huge 1998 physician survey point to a dispirited profession. CMAJ.

[B5] Firth-Cozens J (1998). Individual and organisation predictors of depression in general practitioners. British Journal of General Practice.

[B6] Calnan M, Wainwright D, Forsythe M, Wall B (2000). Health and related behaviour within general practice in South Thames.

[B7] Shearer S, Toedt M (2001). Family physicians' observations of their practice, well being, and health care in the United States. Journal of Family Practice.

[B8] Payne R, Firth-Cozens J, Payne R (1999). Stress at work: a conceptual framework. Stress in health professionals: psychological and organisation causes and interventions.

[B9] McKinstry B, Porter M, Wrate R, Elton R, Shaw J (2004). The MAGPI (Morale Assessment in General Practice Index): A new way for doctors to self-assess their morale. Education for Primary Care.

[B10] The General Practice Assessment Questionnaire. http://www.gpaq.info/Version%202%20GPAQ%20files%20for%202006-7/Consultation%20final%20versions/GPAQ%20Consultation%20V2.pdf.

[B11] Ramsay J, Campbell J, Schroter S, Green J, Roland M (2000). The General Practice Assessment Survey (GPAS): tests of data quality and measurement properties. Fam Pract.

[B12] Bower P, Mead N, Roland M (2002). What dimensions underlie patient responses to the General Practice Assessment Survey? A factor analytic study. Fam Prac.

[B13] Howie JGR, Heaney D, Maxwell M, Walker JJ, Freeman GK, Rai H (1999). Quality at general practice consultations: cross sectional survey. BMJ.

[B14] The General Practice Assessment Questionnaire. National benchmarks for the 2004–5 contract year. http://www.gpaq.info/benchmarks%20consultation%202005_6.htm.

[B15] Mercer SW, Howie JGR (2006). CQI-2, a new measure of holistic interpersonal care in primary care consultations. BJGP.

[B16] McKinstry B, Colthart I, Walker J (2006). Can doctors predict patients' satisfaction and enablement? A cross-sectional observational study. Family Practice.

[B17] Goldberg DP, Hiller VF (1979). A scaled version of the General Health Questionnaire. Psychological Medicine.

[B18] Hasegawa H, Reilly D, Mercer SW, Bikker AP (2005). Holism in primary care: the views of Scotland's general practitioners. Primary Health Care Research and Development.

[B19] Huby G, Gerry M, McKinstry B, Porter M, Shaw J, Wrate R (2002). Morale among general practitioners: Qualitative study exploring relations between partnership arrangements, personal style, and workload. BMJ.

