# Supplementary material for: The impact of general practitioner morale on patient satisfaction with care: a cross-sectional study
Source: BMC Fam Pract. 2007 Sep 28;8:57. doi: 10.1186/1471-2296-8-57 (PMC2072947; doi:10.1186/1471-2296-8-57)
Supplement: Additional file 1 — Magpi new. Example of questionnaire used in this study. [file 1471-2296-8-57-S1.doc]

***Appendix 1***

***MAGPI***

*Please indicate which statement in each of the following groups best reflects how*

*you feel about yourself and your job* ***in the past month:***

|  | **Scoring** | Score |
| --- | --- | --- |
| 1 (a) I feel in control of my work | 1 |  |
| (b) Sometimes I find it hard to manage my work | 2 |  |
| (c) I am having great difficulty managing my workload | 3 |  |
| 2 (a) I have no problems with any of my partners | 1 |  |
| (b) I have some problems with one or more partners | 2 |  |
| (c) I have serious problems with one or more partners | 3 |  |
| 3 (a) I am more up to date with modern general practice than the majority of doctors | 1 |  |
| (b) I am as up to date as most doctors with modern general practice | 2 |  |
| (c) I have not kept up to date with modern practice | 3 |  |
| 4 (a) I feel well supported by the people who work with me | 1 |  |
| (b) Sometimes I feel a bit unsupported by the people who work  with me | 2 |  |
| 1. I don’t feel at all supported by the people who work with   me | 3 |  |
| 5 (a) I have no worries about my health | 1 |  |
| (b) I have only minor worries about my health | 2 |  |
| (c) I have been quite worried about my health | 3 |  |
| 6 (a) I am well supported at home | 1 |  |
| (b) I could be better supported at home | 2 |  |
| (c) I have little support at home | 3 |  |
| 7 (a) I can keep my home life and work in balance satisfactorily | 1 |  |
| 1. It sometimes find it difficult to keep a balance between   work and home life | 2 |  |
| 1. I am finding it very difficult to keep a balance between   work and home life | 3 |  |

| 8 (a) I am a happy person at the moment | 1 |  |
| --- | --- | --- |
| (b) I feel OK but there have been happier times in my life | 2 |  |
| (c) I am unhappy a lot of the time | 3 |  |
| 9 (a) I have family or friends I can turn to | 1 |  |
| (b) I don’t always feel I can turn to friends or family | 2 |  |
| (c) There is no-one I can turn to for help | 3 |  |
| 10 (a) I believe my patients think I do a good job for them | 1 |  |
| (b) I am not sure what my patients think of the job I do for them | 2 |  |
| (c) I believe my patients do not value the job I do | 3 |  |
| 11 (a) I believe my colleagues generally value me | 1 |  |
| (b) I don’t know how my colleagues view me | 2 |  |
| (c) I don’t believe my colleagues value me much | 3 |  |
| 12 (a) I have no problems with alcohol or other drugs | 1 |  |
| (b) I occasionally wonder if I have become too reliant on  alcohol or other drugs alcohol | 2 |  |
| (c) I am worried about my use of alcohol or other drugs | 3 |  |
| 13 (a) I know that I’ve chosen the right career | 1 |  |
| (b) I sometimes wish I had chosen a different career | 2 |  |
| (c) I really regret having chosen my career | 3 |  |
| 14 (a) I have no particular worries about my family at the  moment | 1 |  |
| (b) I have some worries about my family at the moment | 2 |  |
| (c) I have serious worries about my family at the moment | 3 |  |
| Total score |  |  |
